# Identification of Novel Prognostic Biomarkers for Colorectal Cancer by Bioinformatics Analysis

**DOI:** 10.5152/tjg.2024.23264

**Published:** 2024-01-01

**Authors:** Chao Niu, Xiaogang Li, Xian Lei Luo, Hongwei Wan, Wendi Jin, Zhiping Zhang, Wanfu Zhang, Bo Li

**Affiliations:** Department of General Surgery, The Affiliated Hospital of Yunnan University, Kunming, Yunnan Province, China

**Keywords:** Bioinformatics analysis, colorectal cancer, differentially expressed genes, overall survival

## Abstract

**Background/Aims:**

Colorectal cancer (CRC) ranks third among malignancies in terms of global incidence and has a poor prognosis. The identification of effective diagnostic and prognostic biomarkers is critical for CRC treatment. This study intends to explore novel genes associated with CRC progression via bioinformatics analysis.

**Materials and Methods:**

Dataset GSE184093 was selected from the Gene Expression Omnibus database to identify differentially expressed genes (DEGs) between CRC and noncancerous specimens. Functional enrichment analyses were implemented for probing the biological functions of DEGs. Gene Expression Profiling Interactive Analysis and Kaplan–Meier plotter databases were employed for gene expression detection and survival analysis, respectively. Western blotting and real-time quantitative polymerase chain reaction were employed for detecting molecular protein and messenger RNA levels, respectively. Flow cytometry, Transwell, and CCK-8 assays were utilized for examining the effects of *GBA2* and *ST3GAL5* on CRC cell behaviors.

**Results:**

There were 6464 DEGs identified, comprising 3005 downregulated DEGs (dDEGs) and 3459 upregulated DEGs (uDEGs). Six dDEGs were significantly associated with the prognoses of CRC patients, including *PLCE1*, *PTGS1*, *AMT*, *ST8SIA1*, *ST3GAL5,* and *GBA2*. Upregulating *ST3GAL5* or *GBA2* repressed the malignant behaviors of CRC cells.

**Conclusion:**

We identified 6 genes related to CRC progression, which could improve the disease prognosis and treatment.

**Main Points**

- The top-ranked Kyoto Encyclopedia of Genes and Genomes pathway for the upregulated genes is cell cycle, and that for the downregulated genes is metabolic pathways.
- Six downregulated genes are significantly related to patients’ overall survival, including *PLCE1*, *PTGS1*, *AMT*, *ST8SIA1*, *ST3GAL5,* and *GBA2*.
- Overexpressing *ST3GAL5* or* GBA2* suppresses the malignant behaviors of colorectal cancer cells.

## Introduction

Colorectal cancer (CRC) ranks third among malignancies in terms of global incidence and is most commonly observed between 40 and 50 years of age.^1^ Colorectal cancer accounts for almost 50% of all incident cases in men and is the second primary cause of mortality for cancer in the United States.^2^ In China, estimates from 2022 showed that there were approximately 592 232 new CRC cases and 309 114 deaths attributed to the disease.^3^ Colorectal cancer is usually asymptomatic at the early stage; the 5-year survival rate is over 90% at the early stage but is only approximately 12% at advanced stages.^4^ Most patients receive a diagnosis of CRC at advanced stages, when the optimal opportunity for treatment has passed, due to a lack of effective diagnostic approaches.^5^ Despite the advances in techniques, the prognosis for CRC patients diagnosed with metastasis is far from satisfactory.^6^ Hence, for early detection and prognosis prediction of CRC, identifying novel biomarkers is urgently needed.

With the development of sequencing technologies and large-scale sequence research, numerous sequencing data sets have been collected in various databases. Bioinformatics analysis is a critical method for comprehensively examining large databases that contain complex genetic information.^7^ Numerous studies have employed bioinformatics analysis to identify and analyze genes involved in cancer pathogenesis and progression. The findings of differentially expressed genes (DEGs) provide substantial and valuable information for the study of CRC, helping to improve the early detection, therapeutic strategies, and prognosis of CRC.^8^ For example, *COL12A1* and its homologous family proteins such as *COL1A2*, *COL3A1*, and *COL5A1,* are considered prognostic biomarkers for CRC.^9^ Moreover, studies have identified many immune-related prognostic markers that may reflect the immune dysregulation in the tumor microenvironment of CRC.^10^ Despite these findings, CRC remains a complicated disease, and it is still imperative to determine more predictive markers to improve screening and treatment of the disease.

Herein, we selected the dataset GSE184093 from the Gene Expression Omnibus (GEO) database for identifying DEGs between CRC and adjacent non-tumor samples. Functional enrichment analyses were conducted to investigate the potential functional roles of the DEGs, and genes enriched in the top-ranked KEGG pathways were selected for further analyses with bioinformatics tools, aiming to research the molecular mechanisms underpinning CRC progression and to identify potential biomarkers with diagnostic and prognostic values for the disease.

## Materials and Methods

### Data Source

The dataset GSE184093 was obtained from the GEO database (https://www.ncbi.nlm.nih.gov/geo/). ^11^ The platform for this dataset was GPL20115 (Agilent-067406 Human CBC lncRNA + mRNA microarray V4.0), including 9 CRC tissues and 9 noncancerous adjacent tissues.

### Identification of Differentially Expressed Genes

The interactive online tool GEO2R (https://www.ncbi.nlm.nih.gov/geo/geo2r/) was employed for DEG identification between CRC and non-cancerous samples. The volcano plot was generated to depict the results of gene selection. The Benjamini & Hochberg method (false discovery rate) was employed for adjusting the *P*-values. Genes meeting the thresholds of |log2 fold change (FC)| > 0.5 and adjusted *P* (*P *adj.) < .05 were selected as DEGs.

### Functional Enrichment Analysis

To preliminarily investigate the functions of the identified DEGs, Database for Annotation, Visualization, and Integrated Discovery (DAVID) (https://david.ncifcrf.gov/) was employed to conduct Gene Ontology (GO) and KEGG enrichment analyses. The GO analysis included 3 categories, namely, cellular component (CC), molecular function (MF), and biological process (BP). *P* < .05 was set as the threshold. The DEGs enriched in the most significant KEGG pathways were further mapped to obtain the overlapping genes.

### Identification of Overlapping Differentially Expressed Genes

The Gene Expression Profiling Interactive Analysis (GEPIA) database (http://gepia.cancer-pku.cn/) includes 92 rectum adenocarcinoma (READ) samples and 275 colon adenocarcinoma (COAD) samples from The Cancer Genome Atlas (TCGA). DEGs between TCGA-COAD/READ and matched normal samples were downloaded from the GEPIA website using the limma method. The online Venn diagram tool (http://bioinformatics.psb.ugent.be/webtools/Venn/) was utilized to obtain the overlapping DEGs in TCGA-COAD, TCGA-READ, and the most significant KEGG pathways.

### Protein–Protein Interaction Networks

Search Tool for the Retrieval of Interacting Genes, version 11.5 (STRING, https://cn.string-db.org/),^12^ was employed for constructing protein–protein interaction (PPI) networks. Medium confidence score of ≥0.4 was used as the parameter. Gene interactions were represented by nodes and edges. Genes with more interactions with other genes are considered more important.

### Survival Analysis

The DEGs filtered into PPI networks were further analyzed using Kaplan–Meier plotter (https://kmplot.com/analysis/index.php?p=service&cancer=pancancer_rnaseq) to determine their prognostic values in CRC. Kaplan–Meier plotter database contained only RAN-seq data from READ samples (n = 165), not from COAD samples. Log-rank* P *< .05 depicted statistical significance.

### Cell Culture and Transfection

Human normal colon epithelial cell line NCM460 and CRC cell lines (LoVo, HT29, SW480, SW620) were obtained from WheLab (Shanghai, China). LoVo and HT29 were cultured in F-12K medium (WheLab) and MyCoy’s 5A (WheLab), respectively. The SW480, SW620, and NCM460 were incubated in high-glucose Dulbecco’s modified Eagle medium (DMEM; WheLab). Additionally, 10% fetal bovine serum (FBS; Gibco, Grand Island, NY, USA) was supplemented to the media. The cells were cultured at 37°C with 5% CO_2_ in a humidified environment.

To overexpress GBA2 (glucosylceramidase beta 2) or ST3GAL5 (ST3 beta-galactoside alpha-2,3-sialyltransferase 5), GAB2 or ST3GAL5 overexpression vector (GAB2-OE or ST3GAL5-OE) and the control vector were bought from RiboBio (Guangzhou, China). The vectors were separately transfected into CRC cells using Lipofectamine 3000 (Invitrogen, Carlsbad, Calif, USA). Cells were collected after transfection of 48 hours for further experiments.

### Western Blotting

Radioimmunoprecipitation assay buffer (Solarbio, Beijing, China) was employed for protein extraction from cells. Protein concentration was estimated using a bicinchoninic acid assay kit (Beyotime, Shanghai, China). Protein samples were resolved in 10% sodium dodecyl sulfate polyacrylamide gel electrophoresis and blotted onto polyvinylidene fluoride membranes (Beyotime), which were then blocked with 5% defatted milk, incubated with anti-GAPDH (ab9485, 1:2500, Abcam), anti-GBA2 (ab205064, 1:500), and anti-ST3GAL5 (ab155671, 1:1000) primary antibodies (Abcam, Shanghai, China) at 4°C overnight, and washed thrice with Tris-buffered saline in Tween before incubation for 2 hours with the secondary antibody (ab205718, 1:2000, Abcam). Lastly, blot signaling was detected with an ECL detection kit (Solarbio) and estimated by ImageJ software (NIH, Bethesda, Md, USA).

### Real-Time Quantitative Polymerase Chain Reaction 

TRIzol reagent (Invitrogen) was employed for total RNA isolation. Preparation of cDNA was achieved using the iScript cDNA Synthesis Kit (Bio-Rad, Hercules, Calif, USA). Real-time quantitative polymerase chain reaction (RT-qPCR) was conducted on a CFX96 Touch RT-PCR system (Bio-Rad) with SYBR® Premix Ex Taq™ II (Takara, Dalian, China). With normalization to GAPDH, the 2^−^^ΔΔCt^ method was employed for determining relative gene expression. Primers are shown in Table 1.

### Flow Cytometry

An Annexin V-FITC/PI Apoptosis Detection Kit (Beyotime) was utilized for apoptotic cell detection. In short, cells were washed with phosphate-buffered saline, resuspended in binding buffer (Beyotime), followed by treatment with annexin V-FITC (5 μL) and propidium iodide (10 μL) without light for 10 minutes. Apoptotic cells were detected using a flow cytometer (FACScan, BD Biosciences, Franklin, NJ, USA).

### Cell Counting Kit-8 Assay

Colorectal cancer cells (2 × 10^3^/well) were inoculated into 96-well plates and incubated at 37°C for the indicated time period. Afterwards, each well was added with CCK-8 reagent (10 μL, Beyotime) before additional 2-hour culture. The 450 nm optical density was estimated with a microplate reader (Bio-Rad).

### Transwell Assay

A Transwell chamber (24-well, 8-μm pore size; Corning Inc., Corning, NY, USA) was employed for examining tumor cell invasion and migration. For evaluation of invasiveness, the upper chamber was precoated with Matrigel (100 μL; Beyotime), and CRC cells (1 × 10^4^) in serum-free medium (200 μL) were added. The bottom plates were added with complete medium (500 μL) with 10% FBS. After 24 hours, cells that remained in the upper side were gently wiped off using cotton swabs, while those invaded to the bottom plates were dyed with crystal violet for 10 minutes after fixing in 4% paraformaldehyde. For the evaluation of migration, the upper side was not precoated with Matrigel, and other procedures were similar to those of the invasion assay. The results were depicted with a microscope, and 5 randomly selected fields were used to count invaded and migrated cells.

### Statistical Analysis

Data are expressed as mean ± standard deviation. Each experiment was repeated at least 3 times. Difference comparisons between groups were performed by the Student’s *t*-test or one-way analysis of variance, followed by Tukey’s post hoc analysis using GraphPad Prism 8.0.2 software (GraphPad, San Diego, Calif, USA). A *P* < .05 indicated statistical significance.

## Results

### Differentially Expressed Genes

**Figure 1.**
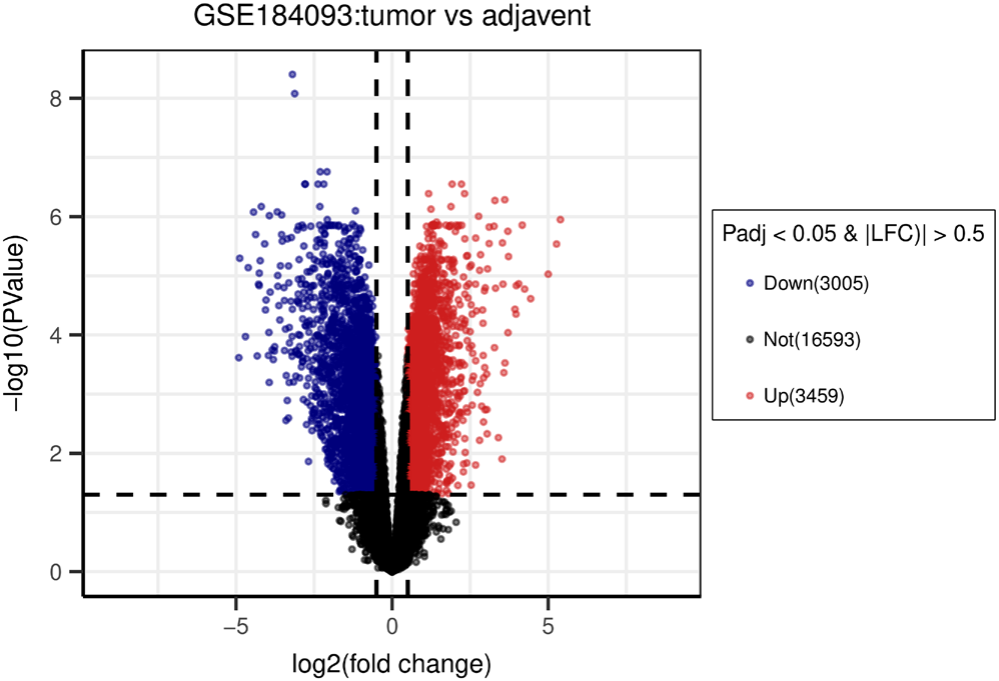
Identified differentially expressed genes (DEGs). Volcano plot of DEGs between normal and CRC specimens from dataset GSE184093. Blue dots: significantly downregulated genes; red dots: significantly upregulated genes; gray dots: not significantly expressed genes. |log2FC| > 0.5 and adjusted *P* < .05 were considered statistically significant. CRC, colorectal cancer.

The dataset GSE184093 was selected for identifying significant DEGs between CRC and noncancerous samples. With the thresholds of *P *adj. < .05 and |log2FC| > 0.5, we identified 6464 DEGs, including 3005 downregulated DEGs (dDEGs) and 3459 upregulated DEGs (uDEGs) (Figure 1).

### Functional Enrichment Analysis

Gene Ontology and KEGG enrichment analyses were performed on the 6464 DEGs to determine their possible functions. The top 5 significant terms and pathways enriched by the uDEGs and dDEGs were screened out. As displayed in Table 2, the uDEGs were mainly enriched in cell division, rRNA processing, DNA replication, cytoplasmic translation, and mitotic sister chromatid segregation in the BP category; nucleus, cytosol, nucleoplasm, kinetochore, and cytosolic ribosome in the CC category; and protein binding, RNA binding, ATPase activity, structural constituent of ribosome, and DNA replication origin binding in the MF category. As for the dDEGs, they were significantly enriched in signal transduction, adaptive immune response, B cell receptor signaling pathway, phagocytosis, and engulfment in the BP category; plasma membrane and so on in the CC category; and antigen binding, immunoglobulin receptor binding, and beta-amyloid binding in the MF category.

Moreover, as depicted by KEGG results, the uDEGs were markedly enriched in cell cycle, ribosome, spliceosome, nucleocytoplasmic transport, and DNA replication, while the dDEGs were predominantly enriched in metabolism-linked pathways like metabolic pathways, the cAMP signaling pathway, and fatty acid degradation. In general, the most significantly enriched KEGG pathway for the uDEGs was cell cycle, and that for the dDEGs was metabolic pathways, with 52 (Supplementary Table 1) and 275 genes (Supplementary Table 2) enriched, respectively.

### Identification of the Overlapping Differentially Expressed Genes

**Figure 2.**
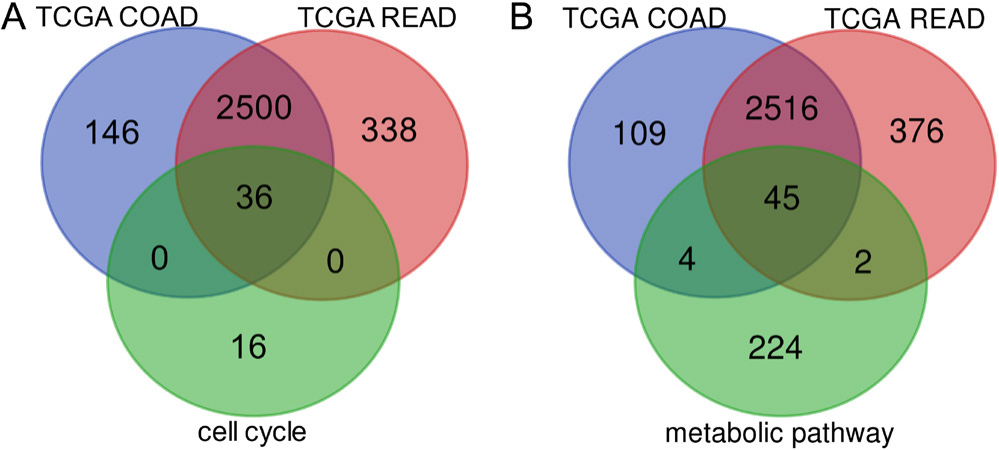
Venn diagrams of DEGs. (A) Upregulated overlapping DEGs. (B) Downregulated overlapping DEGs. Dysregulated genes in COAD or READ were obtained from GEPIA. COAD, colon adenocarcinoma; DEGs, differentially expressed genes; READ, rectal adenocarcinoma; TCGA, The Cancer Genome Atlas.

Using GEPIA database, 5356 and 5813 DEGs were obtained from TCGA-COAD and TCGA-READ, respectively, including 2682 uDEGs and 2674 dDEGs in COAD (Figure 2A and 2B), as well as 2874 uDEGs and 2939 dDEGs in READ (Figure 2A and 2B). The web tool Venn diagrams was utilized to identify the overlapping DEGs from the 3 cohorts: COAD, READ, and cell cycle/metabolic pathways. As a result, 36 of the 52 genes that were enriched in cell cycle were validated to be upregulated in both COAD and READ samples, and 45 of the 275 genes enriched in metabolic pathways were validated to be downregulated.

### Construction of Protein–Protein Interaction Networks

**Figure 3.**
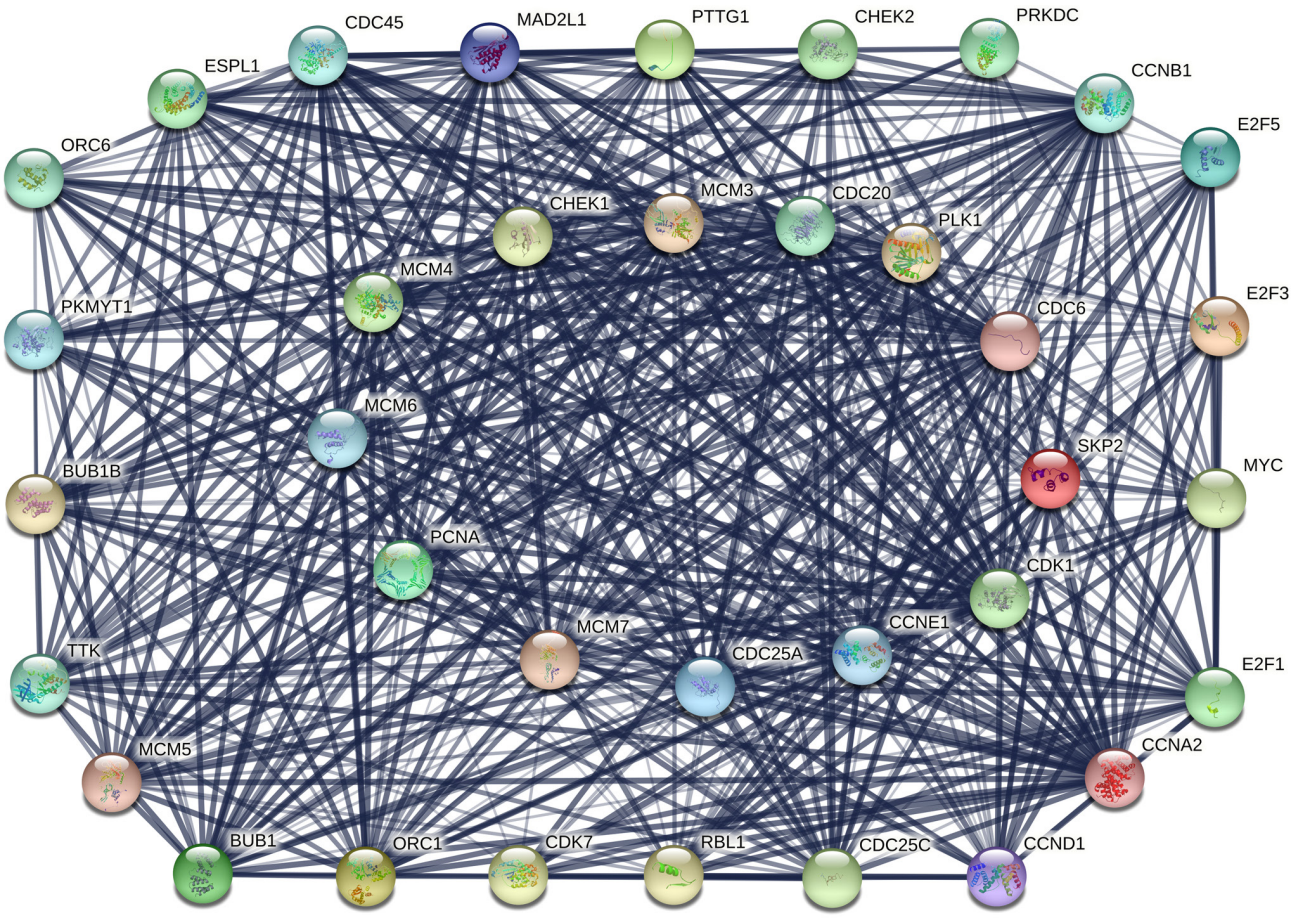
Protein–protein interaction network for the overlapping upregulated differentially expressed genes.

**Figure 4.**
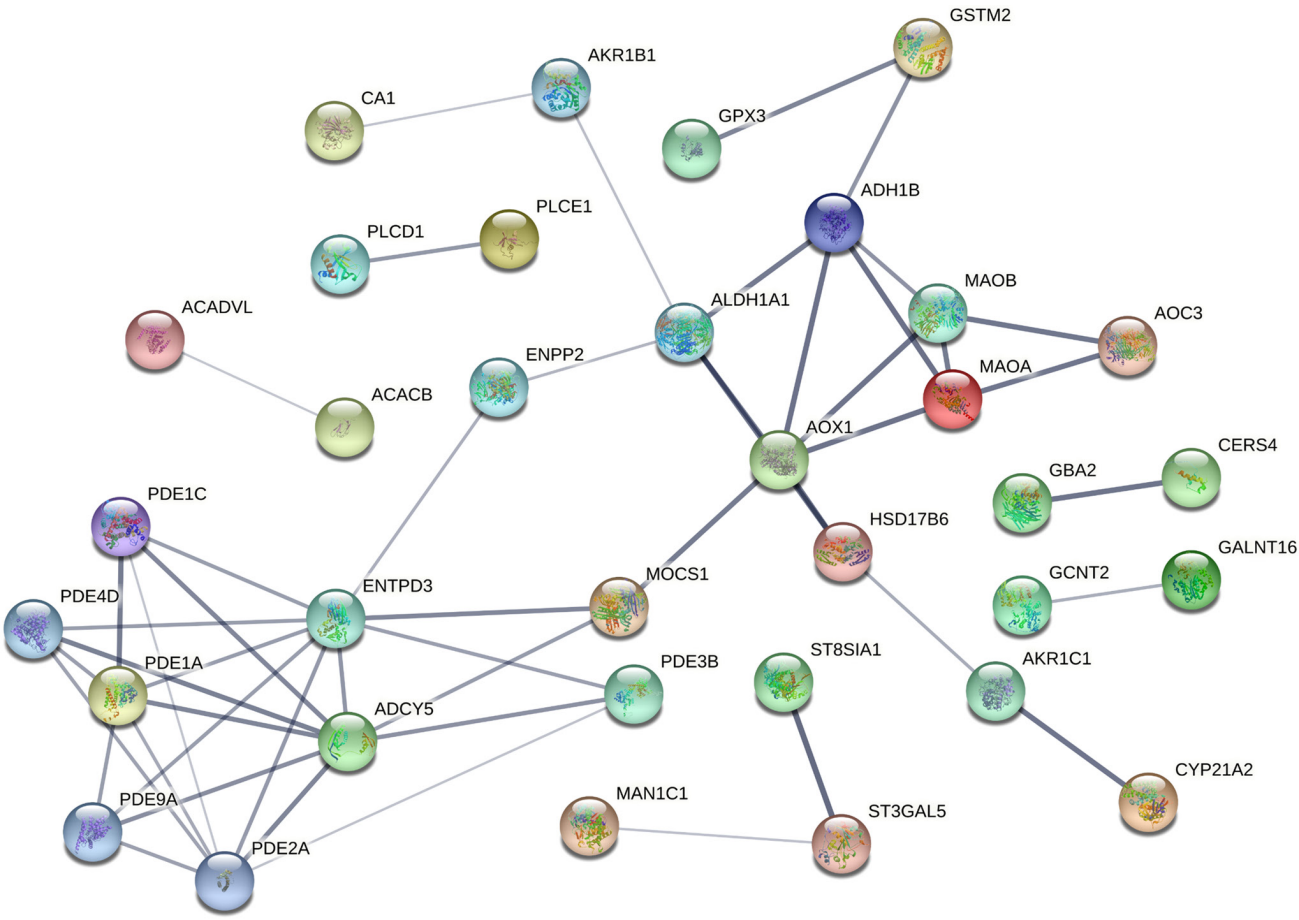
Protein–protein interaction network for the overlapping downregulated differentially expressed genes.

The PPI networks were constructed using the STRING online database to investigate the interactions among the overlapping DEGs. As shown by the results, the PPI network for the overlapping uDEGs contained 36 nodes and 527 edges (Figure 3), while that for the overlapping dDEGs consisted of 34 nodes and 50 edges (Figure 4).

### Validation of Gene Prognostic Value

**Figure 5.**
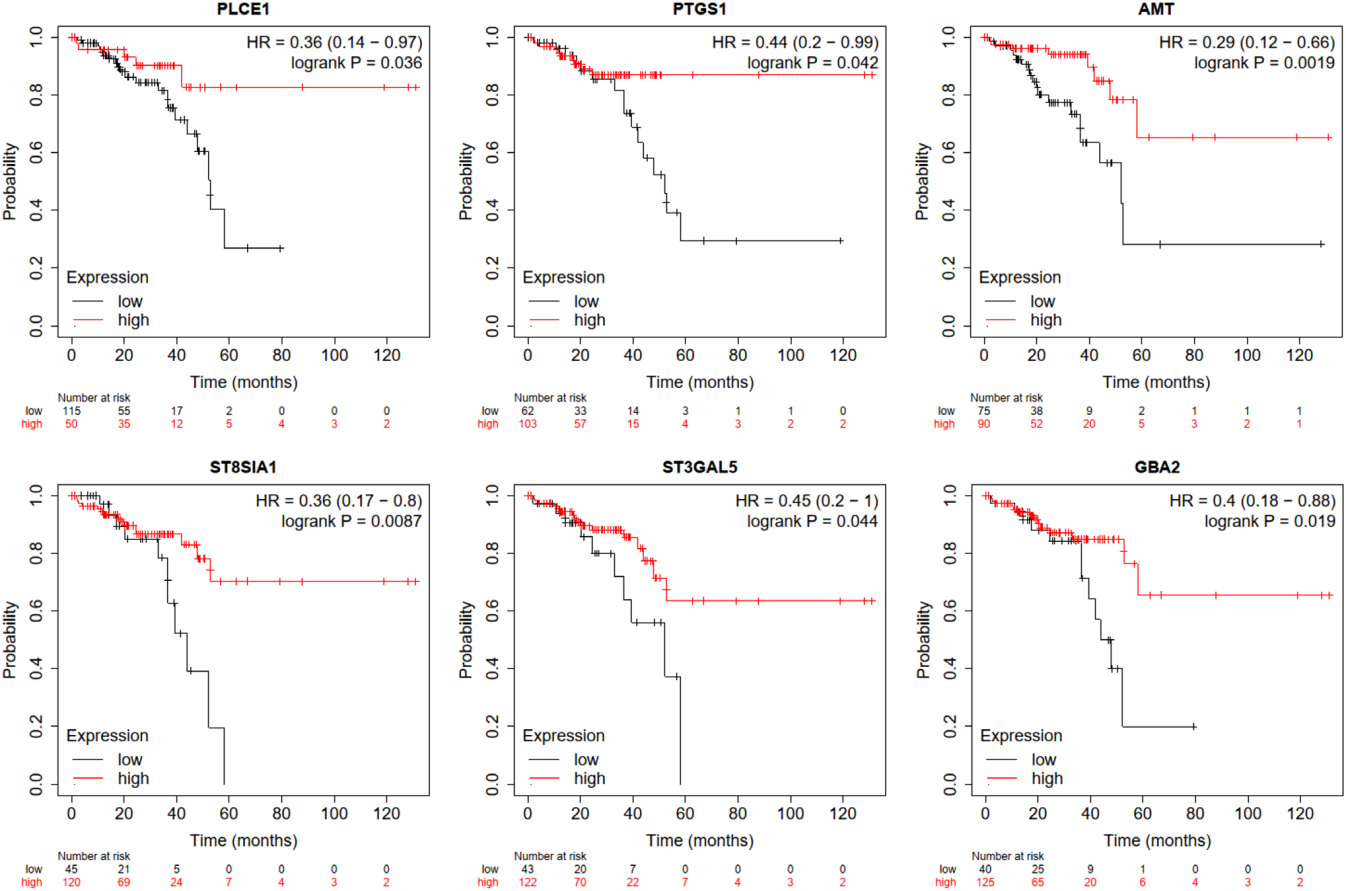
Survival analysis. Kaplan–Meier plotter database for analyzing the correlation between gene expression and overall survival of rectal adenocarcinoma patients.

Next, we further verified the prognostic values of these nodes/DEGs using Kaplan–Meier plotter database. The samples were segregated into high- and low-expression groups based on the gene median value. Notably, the mRNA levels of 6 dDEGs were prominently associated with READ patients’ prognosis, including phospholipase C epsilon 1 (*PLCE1*), cyclooxygenase 1 (*PTGS1)*, aminomethyltransferase (*AMT*), ST8 alpha-*N*-acetyl-neuraminide alpha-2,8-sialyltransferase 1 (*ST8SIA1*), *ST3GAL5*, and *GBA2*. A lower mRNA level of the 6 genes predicted a worse overall survival of patients (Figure 5).

### Validation of Gene Expression in Colorectal Cancer Cell Lines

**Figure 6.**
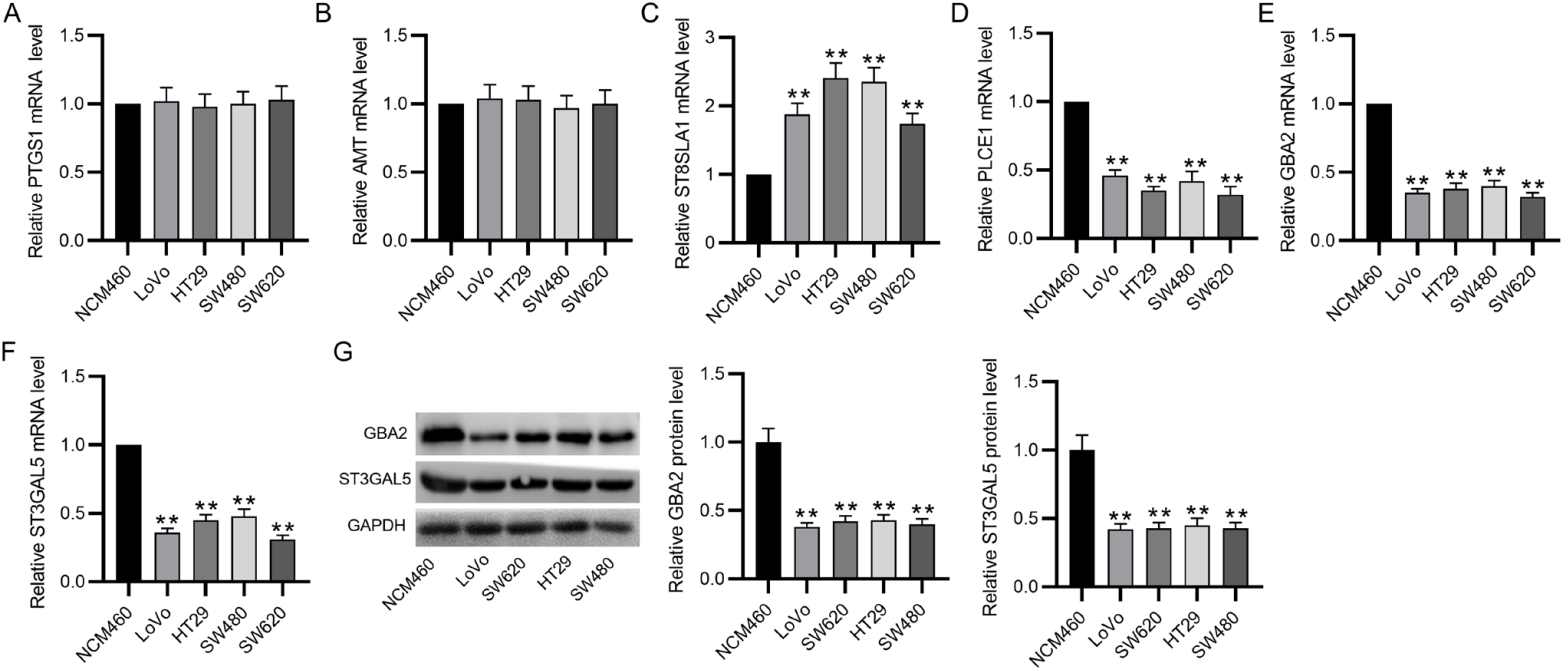
Validation of gene expression in CRC cell lines. (A-F) RT-qPCR analysis of mRNA levels of *PLCE1*, *PTGS1*, *AMT*, *ST8SIA1*, *GBA2,* and* ST3GAL5 *in CRC cell lines and the normal human colon epithelial cell line NCM460. (G) Western blotting for evaluating protein levels of GBA2 and ST3GAL5 in different cell lines. CRC, colorectal cancer; RT-qPCR, real-time quantitative polymerase chain reaction. **P < .01.

Subsequently, RT-qPCR was conducted to detect gene expression in CRC cell lines. As depicted by the results, there was no marked difference in *PTGS1* and *AMT *expression between tumor and normal cell lines (Figure 6A and 6B). The *ST8SIA1* mRNA level in CRC cells was prominently higher than that in NCM460 cells (Figure 6C), which was in conflict with the microarray analysis. Moreover, the mRNA levels of *PLCE1*, *GBA2*, and *ST3GAL5* were markedly reduced in CRC cell lines (Figure 6D-F), which were consistent with the microarray results. Additionally, *PLCE1* has previously been shown to suppress the malignancy of CRC cells.^13^ Therefore, *GBA2 *and *ST3GAL5* were selected for further analyses. Similar to RT-qPCR, western blotting displayed significantly decreased levels of GBA2 and ST3GAL5 proteins in CRC cell lines (Figure 6G).

### The Effect of Gene Overexpression on Colorectal Cancer Cell Malignancy

**Figure 7.**
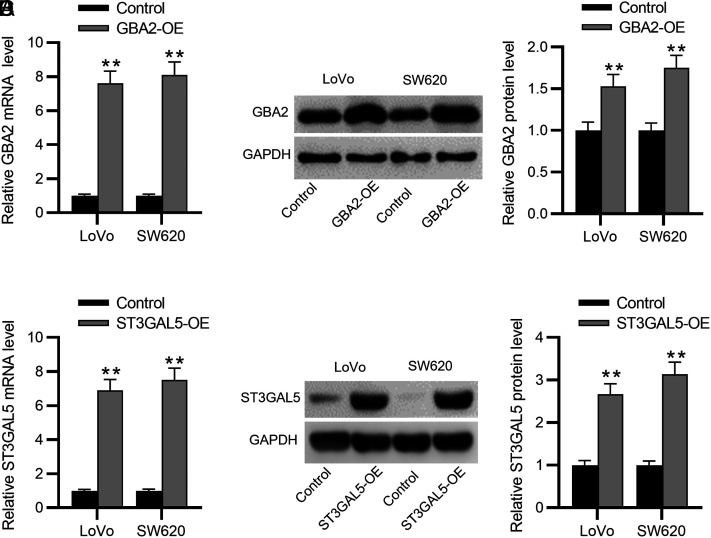
Overexpression efficiency of GBA2 or ST3GAL5 in CRC cells. RT-qPCR (A) and western blotting (B) for detecting GBA2 mRNA and protein expression in CRC cells with/without GBA2-OE transfection. RT-qPCR (C) and western blotting (D) for detecting ST3GAL5 mRNA and protein expression in CRC cells with/without ST3GAL5-OE transfection. CRC, colorectal cancer; mRNA, messenger ribonucleic acid; RT-qPCR, real-time quantitative polymerase chain reaction. **P < .01.

**Figure 8.**
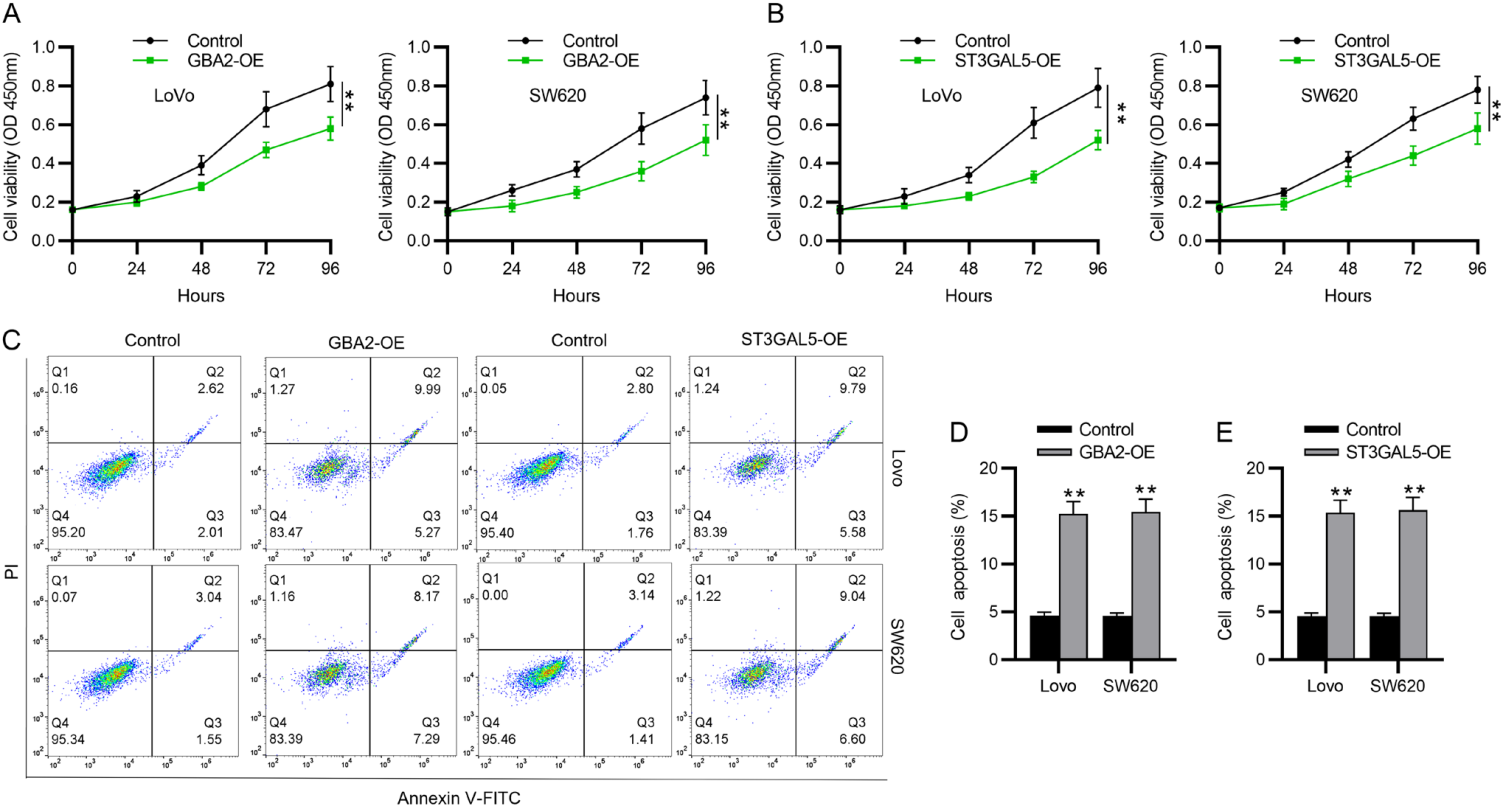
Upregulating GBA2 or ST3GAL5 impacts CRC cell viability and apoptosis. CCK-8 assay showing CRC cell viability with/without upregulation of (A) GBA2 or (B) ST3GAL5. (C-E) Flow cytometry for detecting apoptotic CRC cells in different treatment groups. CRC, colorectal cancer. **P < .01.

**Figure 9.**
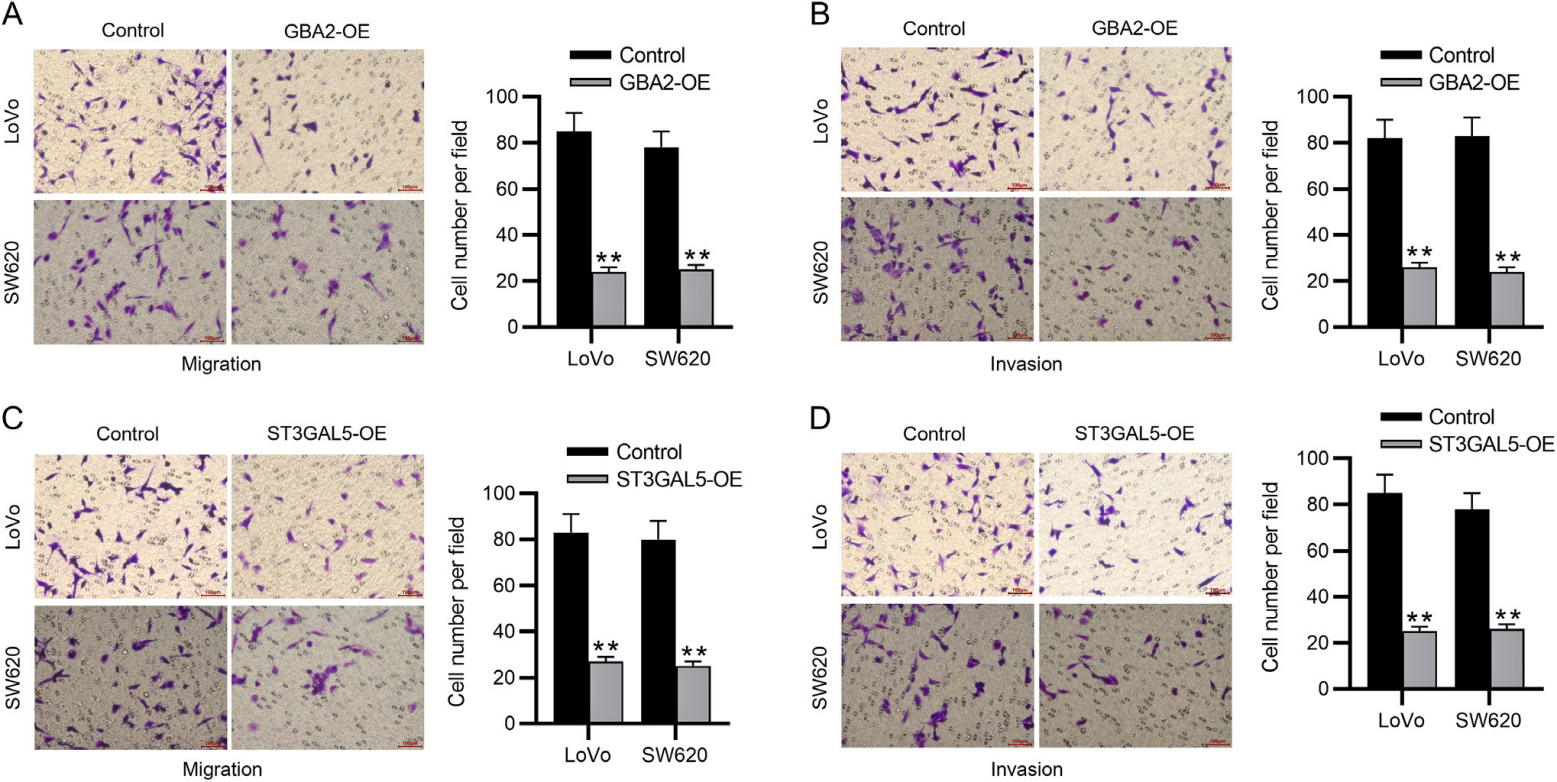
Overexpressing GBA2 or ST3GAL5 affects CRC cell motility. Transwell assays for detecting CRC cell migration (A) and invasion (B) with/without GBA2 upregulation. Transwell assays for detecting CRC cell migration (C) and invasion (D) with/without ST3GAL5 upregulation. CRC, colorectal cancer. **P < .01.

To determine the specific roles of *GBA2* and *ST3GAL5* in affecting the biological behaviors of CRC cells, we separately overexpressed *GBA2* and *ST3GAL5* in 2 CRC cell lines (LoVo and SW620) using their corresponding overexpression vectors (GBA2-OE or ST3GAL5-OE). The transfection of GBA2-OE resulted in an elevation in GBA2 expression in CRC cells (Figure 7A and 7B). Similar results were shown in CRC cells with ST3GAL5-OE transfection (Figure 7C and 7D). These data confirmed that *GBA2* and *ST3GAL5* were successfully overexpressed in CRC cells. Then, functional assays were implemented to estimate whether *GBA2* or *NANA* overexpression impacted CRC cell phenotypes. As depicted by CCK-8 assay, GBA2-OE or ST3GAL5-OE markedly reduced the viability of both LoVo and SW620 cells (Figure 8A and 8B). In parallel, flow cytometry analysis demonstrated that overexpressing *GBA2* or *ST3GAL5* contributed to cell apoptosis (Figure 8C and 8E). Furthermore, Transwell assays revealed that GBA2-OE remarkably impaired the migration and invasiveness of CRC cells (Figure 9A and 9B). Additionally, ST3GAL5-OE exhibited similar effects to GBA2-OE on CRC cell migration and invasiveness (Figure 9C and 9D). Collectively, GBA2 or ST3GAL5 upregulation restrains the malignant behavior of CRC cells.

## Discussion

In the present study, we identified 3459 uDEGs and 3005 dDEGs between normal and CRC samples from the dataset GSE184093. Functional enrichment analysis depicted that the majority of these genes were prominently related to cell cycle and metabolic pathways, respectively (Table 2). Subsequently, the GEPIA database was utilized to validate the DEGs enriched in cell cycle and metabolic pathways. As a result, we identified 36 uDEGs and 45 dDEGs (Figure 2) and then constructed PPI networks of these DEGs, which revealed the interaction between different proteins and helped to screen out core regulatory genes. The survival analysis revealed that 6 of the DEGs filtered into PPI networks were prominently associated with CRC patients’ prognosis, including *PTGS1*, *AMT*, *PLCE1*, *ST8SIA1*, *ST3GAL5,* and *GBA2* (Figure 5). The RT-qPCR analysis showed that *PLCE1*, *ST8SIA1*, *ST3GAL5*, and *GBA2* were aberrantly regulated between the tumor and nontumor cell lines, while the results of *PTGS1* and *AMT* showed no significance (Figure 6). In vitro experiments showed that overexpressing *ST3GAL5* or *GBA2* suppressed the malignant behaviors of CRC cells (Figures 8 and 9).

The DEG *PTGS1*, also known as cyclooxygenase 1 (*COX-1*), encodes an enzyme catalyzing arachidonate conversion to prostaglandin. Several studies have shown that *PTGS1*/*COX1* is upregulated in CRC tissues, and inhibition of *COX-1* can suppress CRC cell aggressiveness.^14^ Contrarily, *PTGS1*/*COX1* downregulation in CRC was also reported,^15^ supporting the microarray results in this study. The DEG *AMT* (aminomethyltransferase) is a component of the glycine cleavage system termed T-protein. Mutations in this gene in humans lead to nonketotic hyperglycinemia.^16^ Proteomic analysis reveals that AMT protein expression can be upregulated by the effect of retinoic acids in breast cancer cells, which is associated with tumor cell apoptosis.^17^ Here, we identified that *AMT* was downregulated in CRC specimens and that its higher level was significantly linked to better overall survival in CRC patients. In addition, among the 6 DEGs identified, *AMT* has the best 95% CI range (0.12–0.66) in the survival analysis, indicating its potential significance in CRC prognosis. However, genes *PTGS1* and *AMT* were not validated at the cell line level, which may be ascribed to the limitations of cell lines, highlighting the need for further research.

The phospholipase C epsilon 1 gene, encoding a phospholipase enzyme, catalyzes phosphatidylinositol-4,5-bisphosphate hydrolysis to produce two secondary messengers: diacylglycerol (DAG) and inositol 1,4,5-triphosphate (IP3). Inositol 1,4,5-triphosphate and DAG subsequently regulate various processes that affect gene expression, cell growth, and differentiation.^18^ Evidence indicates that *PLCE1* is tightly related to the clinical staging and survival of CRC patients.^19^ Importantly, *PLCE1* has been indicated to exert an inhibitory effect on the incidence of CRC. *PLCE1* is lowly expressed in CRC samples, and its overexpression markedly suppresses CRC cell proliferation,^13^ which was consistent with our results. *ST8SIA1* encodes a type II membrane protein catalyzing the synthesis of ganglioside GD3, which is critical for cell growth and adhesion of cultured tumor cells.^20^ Previous research has demonstrated the involvement of *ST8SIA1* in several cancers. For example, in bladder cancer, *ST8SIA1* suppresses cell proliferative, invasive, and migratory capabilities by inhibiting the JAK/STAT3 pathway.^21^ *ST8SIA1* knockout dramatically blocks tumor metastasis and growth of triple-negative breast carcinoma via the FAK-AKT-mTOR signaling pathway.^22^ These indicate that *ST8SIA1* may work as a tumor activator or suppressor depending on cancer types. Intriguingly, evidence has demonstrated *ST8SIA1* upregulation in CRC tissues and that upregulating *ST8SIA1* could attenuate miR-33a/let-7e-mediated suppressive effects on cell proliferation and chemoresistance in CRC.^23^ Consistent with this, our experimental data depicted that the *ST8SIA1 *level in CRC cell lines was much higher than in the nontumor cell line.

The *ST3GAL5* gene also encodes a type II membrane protein that converts lactosylceramide into GM3. *ST3GAL5 *has been verified to be implicated in multiple malignancies, such as bladder cancer and hepatocellular carcinoma.^24,25^ Previous evidence has suggested that *ST3GAL5* is overexpressed in rectal carcinoma samples compared to normal samples.^26^ On the contrary, this study revealed that *ST3GAL5 *was underexpressed in CRC samples and that overexpressing *ST3GAL5* led to reduced aggressiveness of CRC cells in vitro. *GBA2* encodes a microsomal beta-glucosidase catalyzing the hydrolysis of bile acid 3-O-glucosides as endogenous compounds. *GBA2* can mitigate the tumorigenicity of human melanoma cells.^27^ However, the expression and role of *GBA2* in CRC have never been studied. Similar to previous reports, our study depicted that *GBA2* upregulation repressed the malignant behaviors of CRC cells, indicating that *GBA2* might work as a tumor suppressor in CRC. In addition, functional enrichment analysis showed that both *ST3GAL5* and *GBA2* were enriched in metabolic pathways. This suggests that overexpression of these two genes may suppress the malignant behaviors of CRC by regulating metabolism-related pathways.

It is worth noting that our study has some limitations. First, differential gene analysis was conducted based on a single dataset, which may result in selection bias. More public datasets are needed for a comprehensive bioinformatics analysis. Second, laboratory analyses for *AMT* were lacking, and future studies would benefit from further data concerning this gene. Moreover, we only conducted in vitro experiments for verification. Data from in vivo experiments would help to elucidate the findings.

In conclusion, we identified 6 DEGs that might be implicated in the pathogenesis and prognosis of CRC via bioinformatics analysis, including *PLCE1*, *PTGS1*, *AMT*, *ST8SIA1*, *ST3GAL5,* and *GBA2*. Moreover, overexpressing *GBA2 *or* ST3GAL5* attenuated CRC cell malignancy in vitro. Our results may provide new clues for improving the detection and prediction of CRC.

## Figures and Tables

**Table 1.** Primer Sequences Used for Real-Time Quantitative Polymerase Chain Reaction

Gene	Forward (5’–3’)	Reverse (5’–3’)
PLCE1	CCTGCCAATAATGAGGAAGAG	CAGAATGTTGTCATCAGAAAGG
PTGS1	ATGGAGACAATCTGGAGCG	TTTCTCCATCCAGCACCTG
AMT	AGCTAAGACCAAACCAGGG	TGACCTTGTCCTGCATGAG
ST8SIA1	GAATACACTAAGGATGTTGGATCC	AAGGTTCTGAAACCTTTGCC
GBA2	GGACCCAGATGATGAACCA	TTCAGGTTCAGGTCCTTCC
ST3GAL5	CTTCCCTGCAATGGTACAC	GCAATGTACATTTGAGGATGTC
GAPDH	GGAGCGAGATCCCTCCAAAAT	GGCTGTTGTCATACTTCTCATGG

**Table 2.** Functional Enrichment Analysis of Differentially Expressed Genes

Category	Term	Count	*P*
Upregulated genes			
GOTERM_BP_DIRECT	GO:0051301~cell division	120	1.47 × 10^−24^
GOTERM_BP_DIRECT	GO:0006364~rRNA processing	58	1.62 × 10^−20^
GOTERM_BP_DIRECT	GO:0000070~mitotic sister chromatid segregation	24	3.60 × 10^−^^14^
GOTERM_BP_DIRECT	GO:0006260~DNA replication	47	5.84 × 10^−14^
GOTERM_BP_DIRECT	GO:0002181~cytoplasmic translation	39	1.42 × 10^−13^
GOTERM_CC_DIRECT	GO:0005654~nucleoplasm	714	3.81 × 10^−43^
GOTERM_CC_DIRECT	GO:0005829~cytosol	826	1.09 × 10^−20^
GOTERM_CC_DIRECT	GO:0005634~nucleus	884	1.30 × 10^−^^20^
GOTERM_CC_DIRECT	GO:0000776~kinetochore	52	4.39 × 10^−14^
GOTERM_CC_DIRECT	GO:0022626~cytosolic ribosome	34	6.01 × 10^−12^
GOTERM_MF_DIRECT	GO:0005515~protein binding	1777	5.06 × 10^−28^
GOTERM_MF_DIRECT	GO:0003723~RNA binding	317	5.89 × 10^−26^
GOTERM_MF_DIRECT	GO:0003688~DNA replication origin binding	17	3.05 × 10^−10^
GOTERM_MF_DIRECT	GO:0003735~structural constituent of ribosome	57	9.32 × 10^−10^
GOTERM_MF_DIRECT	GO:0016887~ATPase activity	75	1.93 × 10^−07^
KEGG_PATHWAY	hsa04110:Cell cycle	52	3.21 × 10^−14^
KEGG_PATHWAY	hsa03030:DNA replication	22	3.20 × 10^−10^
KEGG_PATHWAY	hsa03013:Nucleocytoplasmic transport	39	7.06 × 10^−09^
KEGG_PATHWAY	hsa03010:Ribosome	45	1.13 × 10^−06^
KEGG_PATHWAY	hsa03040:Spliceosome	42	2.50 × 10^−06^
Downregulated genes			
GOTERM_BP_DIRECT	GO:0050853~B cell receptor signaling pathway	37	7.89 × 10^−08^
GOTERM_BP_DIRECT	GO:0006911~phagocytosis, engulfment	34	1.05 × 10^−07^
GOTERM_BP_DIRECT	GO:0007165~signal transduction	189	3.08 × 10^−07^
GOTERM_BP_DIRECT	GO:0006955~immune response	87	2.46 × 10^−06^
GOTERM_BP_DIRECT	GO:0002250~adaptive immune response	80	2.55 × 10^−06^
GOTERM_CC_DIRECT	GO:0005886~plasma membrane	754	3.64 × 10^−27^
GOTERM_CC_DIRECT	GO:0043235~receptor complex	63	4.03 × 10^−13^
GOTERM_CC_DIRECT	GO:0005887~integral component of plasma membrane	246	4.54 × 10^−13^
GOTERM_CC_DIRECT	GO:0009897~external side of plasma membrane	101	1.18 × 10^−12^
GOTERM_CC_DIRECT	GO:0070062~extracellular exosome	335	1.01 × 10^−10^
GOTERM_MF_DIRECT	GO:0003823~antigen binding	38	4.75 × 10^−07^
GOTERM_MF_DIRECT	GO:0004714~transmembrane receptor protein tyrosine kinase activity	31	7.66 × 10^−06^
GOTERM_MF_DIRECT	GO:0001540~beta-amyloid binding	25	9.96 × 10^−06^
GOTERM_MF_DIRECT	GO:0045028~G-protein coupled purinergic nucleotide receptor activity	9	1.83 × 10^−05^
GOTERM_MF_DIRECT	GO:0034987~immunoglobulin receptor binding	26	2.56 × 10^−05^
KEGG_PATHWAY	hsa01100:Metabolic pathways	275	8.88 × 10^−12^
KEGG_PATHWAY	hsa04960:Aldosterone-regulated sodium reabsorption	18	5.13 × 10^−07^
KEGG_PATHWAY	hsa00071:Fatty acid degradation	18	6.53 × 10^−06^
KEGG_PATHWAY	hsa00280:Valine, leucine and isoleucine degradation	19	8.44 × 10^−06^
KEGG_PATHWAY	hsa04024:cAMP signaling pathway	51	1.49 × 10^−05^

**Supplementary Table 1.** The uDEGs Enriched in Cell Cycle

symbol	ID	logFC	AveExpr	t	*P*	adj. *P*	B	Regulation
MCM7	A_33_P3258223	1.127731	12.23252	6.693527	2.16E-06	6.59E-05	5.013391	Up
BUB1B	A_23_P163481	1.14406	9.961157	4.462383	.000269	.001954	0.230906	Up
CDC20	A_23_P149200	1.030855	10.85435	5.776644	1.46E-05	.000241	3.112698	Up
CCND2	A_24_P278747	0.681002	13.55027	2.602936	.017504	.048731	-3.8017	Up
PTTG1	A_23_P7636	1.363654	10.75349	4.937012	9.23E-05	.000897	1.286923	Up
CCND1	A_23_P202837	1.648414	11.08445	6.93636	1.33E-06	4.86E-05	5.497844	Up
PTTG2	A_23_P18579	1.382377	10.67977	6.154078	6.57E-06	.000138	3.908301	Up
MYC	A_33_P3245163	1.688663	9.377633	7.715857	2.92E-07	1.74E-05	6.996497	Up
CHEK2	A_33_P3386760	0.984969	8.165533	6.797516	1.75E-06	5.74E-05	5.221859	Up
CHEK1	A_33_P3349536	1.449325	10.4866	5.567009	2.30E-05	.000327	2.663496	Up
SKP2	A_23_P156310	1.247028	13.03664	6.995387	1.18E-06	4.46E-05	5.614354	Up
ANAPC7	p38472_v4	0.570255	9.795781	4.960315	8.76E-05	.00086	1.338442	Up
CDC25C	A_23_P70249	1.03301	6.563148	5.203926	5.10E-05	.000579	1.874512	Up
CDC25A	A_24_P397107	1.234092	7.367031	5.241043	4.70E-05	.000545	1.955749	Up
CDC25B	A_23_P210726	1.605224	9.979942	5.772753	1.48E-05	.000242	3.104405	Up
CCNA2	A_23_P58321	1.265105	12.20277	4.770843	.000134	.001171	0.918521	Up
RBL1	A_23_P28733	1.154919	8.25474	6.710311	2.09E-06	6.45E-05	5.047141	Up
ESPL1	A_23_P32707	0.962837	8.764788	5.773992	1.47E-05	.000242	3.107046	Up
CCNE1	A_23_P209200	1.199235	8.715661	7.622999	3.48E-07	1.97E-05	6.82252	Up
MCM3	A_23_P7873	1.189972	11.27395	6.45738	3.50E-06	9.00E-05	4.534449	Up
MCM4	A_23_P370989	1.359916	11.72091	8.59898	5.78E-08	6.77E-06	8.590046	Up
MCM5	A_33_P3284951	0.603453	10.83657	3.272949	.004014	.015524	-2.4061	Up
MCM6	A_23_P90612	1.146906	9.56326	6.229838	5.61E-06	.000123	4.065837	Up
ANAPC1	A_23_P17204	0.920253	10.59262	6.03176	8.51E-06	.000165	3.652399	Up
ANAPC13	A_32_P16854	0.537013	10.8523	3.896115	.000976	.005214	-1.03481	Up
PCNA	A_33_P3258612	1.193839	11.61722	5.975803	9.58E-06	.000179	3.534702	Up
PRKDC	A_33_P3337599	1.226649	10.74331	6.39388	3.99E-06	9.77E-05	4.404369	Up
TTK	A_23_P259586	1.395785	8.236359	4.54764	.000222	.001699	0.421329	Up
PKMYT1	A_33_P3397443	1.045082	8.153286	5.668608	1.85E-05	.000282	2.881819	Up
ANAPC10	A_23_P250994	0.59944	9.251698	4.006495	.000759	.004277	-0.78856	Up
ANAPC11	A_23_P27147	0.515852	12.41137	4.064975	.000664	.003862	-0.65791	Up
ORC5	A_23_P31414	0.523999	9.854167	3.324922	.003571	.014236	-2.29361	Up
CCNB1	A_23_P122197	1.118872	9.804877	4.13612	.000565	.003416	-0.49884	Up
ORC6	A_23_P100344	1.160469	6.869522	5.399914	3.31E-05	.000427	2.302023	Up
CDC45	A_23_P57379	1.553303	8.61254	5.70528	1.71E-05	.000267	2.960338	Up
ORC1	A_23_P45799	1.050819	6.04932	3.984434	.000798	.004448	-0.83781	Up
ORC3	A_23_P42045	0.61152	10.82832	5.895634	1.14E-05	.000202	3.365404	Up
E2F1	A_23_P80032	1.074514	8.50105	6.314136	4.71E-06	.000109	4.240245	Up
E2F3	A_23_P385034	0.625518	9.577559	4.52433	.000234	.001765	0.369289	Up
E2F4	A_23_P152218	0.692624	12.12424	5.713748	1.68E-05	.000264	2.978448	Up
BUB3	A_23_P320658	0.694705	11.13586	5.721985	1.65E-05	.000262	2.996056	Up
E2F5	A_23_P31721	1.059338	9.307256	7.359835	5.76E-07	2.68E-05	6.322763	Up
BUB1	A_23_P124417	1.449685	10.69896	5.420236	3.17E-05	.000415	2.346137	Up
TGFB2	A_24_P402438	1.582769	9.292814	3.760196	.001331	.006624	-1.33713	Up
PLK1	A_23_P118174	1.320923	12.11083	6.08145	7.66E-06	.000153	3.756583	Up
CDC7	A_23_P148807	1.085258	8.669747	5.693904	1.75E-05	.000272	2.935998	Up
CDC6	A_33_P3218450	0.664513	8.457078	3.953014	.000857	.004721	-0.90794	Up
WEE1	A_33_P3290567	0.638938	8.671859	4.717903	.000151	.001283	0.800808	Up
CDK7	A_23_P133585	0.624274	10.01751	3.524521	.002274	.009971	-1.85778	Up
CDK1	A_23_P138507	1.263913	8.793763	5.055162	7.09E-05	.000734	1.547716	Up
ATR	A_23_P136058	0.819954	9.441672	4.716755	.000151	.001285	0.798256	Up
MAD2L1	A_23_P92441	1.817192	8.97937	6.873679	1.50E-06	5.29E-05	5.373586	Up

**Supplementary Table 2.** The dDEGs Enriched in Metabolic Pathways

symbol	ID	logFC	AveExpr	t	*P*	adj. *P*	B	Regulation
HSD3B2	A_23_P51580	-4.91036	9.710022	-5.77514	1.47E-05	.000241	3.109485	Down
CA1	A_23_P168916	-4.88115	12.45225	-8.94976	3.13E-08	5.04E-06	9.192891	Down
CA4	A_23_P4096	-3.90582	11.51677	-7.65287	3.28E-07	1.89E-05	6.878628	Down
UGT1A5	A_24_P222872	-3.84174	9.562798	-5.98005	9.49E-06	.000178	3.543646	Down
ADH1A	A_24_P291658	-3.66347	10.02596	-8.25402	1.07E-07	9.87E-06	7.980654	Down
AMPD1	A_23_P51787	-3.54053	8.187871	-9.83171	7.12E-09	2.00E-06	10.63602	Down
UGT2A3	A_24_P334378	-3.51902	5.576466	-9.57983	1.08E-08	2.59E-06	10.23421	Down
GBA3	A_23_P18672	-3.44614	7.89973	-5.96203	9.86E-06	.000183	3.505666	Down
SI	A_32_P302205	-3.35884	9.178982	-4.71793	.000151	.001283	0.800874	Down
CA2	A_23_P8913	-3.29318	11.89477	-6.5928	2.65E-06	7.48E-05	4.81003	Down
B3GNT6	A_23_P127978	-3.08253	8.849999	-5.30357	4.10E-05	.000495	2.092323	Down
AKR1B10	A_24_P129341	-2.8407	10.62808	-4.73957	.000144	.001236	0.849006	Down
CYP2C8	A_23_P161368	-2.74009	5.856142	-4.16043	.000534	.003279	-0.44447	Down
HMGCS2	A_23_P103588	-2.73456	12.92734	-5.75379	1.54E-05	.00025	3.063961	Down
PCK1	A_23_P408249	-2.71762	11.81189	-6.74799	1.93E-06	6.14E-05	5.122754	Down
HSD17B2	A_33_P3367037	-2.70854	8.403097	-7.41238	5.21E-07	2.55E-05	6.423335	Down
ADH1B	A_33_P3353737	-2.64118	8.459066	-9.06237	2.58E-08	4.50E-06	9.382867	Down
HPSE2	A_23_P61707	-2.61369	7.54607	-6.0172	8.77E-06	.000169	3.621811	Down
CYP2C9	A_23_P12767	-2.61154	5.757799	-4.59376	.0002	.001574	0.524248	Down
DHRS9	A_23_P56559	-2.57676	10.09206	-4.82752	.000118	.001073	1.044372	Down
PDE6A	A_23_P81590	-2.57457	6.507244	-6.20237	5.94E-06	.000128	4.008813	Down
LDHD	A_23_P54918	-2.54057	10.47796	-8.49558	6.95E-08	7.43E-06	8.40912	Down
MOGAT2	A_33_P3353866	-2.53827	12.09718	-5.46785	2.86E-05	.000384	2.449335	Down
HPGDS	A_23_P10506	-2.34952	8.854698	-6.13666	6.82E-06	.000142	3.871976	Down
CA12	A_24_P330518	-2.34819	10.64656	-5.29855	4.14E-05	.000499	2.081379	Down
PLCD1	A_23_P80739	-2.30298	9.848648	-13.8121	2.44E-11	1.74E-07	16.03076	Down
AKR1C3	A_23_P138541	-2.2978	11.32494	-4.6372	.000181	.001457	0.621098	Down
B3GALT5	A_23_P102919	-2.27846	11.84953	-5.2927	4.19E-05	.000503	2.068609	Down
CKB	A_23_P25674	-2.26101	11.80866	-5.90626	1.11E-05	.000198	3.387887	Down
HSD11B2	A_23_P14986	-2.22789	11.88704	-8.11343	1.39E-07	1.16E-05	7.727509	Down
GDPD3	A_23_P26511	-2.19895	10.32687	-6.29551	4.89E-06	.000112	4.201782	Down
ST6GALNAC1	A_23_P54968	-2.19657	12.6467	-7.89611	2.08E-07	1.45E-05	7.330713	Down
ENTPD5	A_23_P117580	-2.18865	12.5049	-12.903	7.86E-11	2.82E-07	14.94098	Down
GCNT3	A_23_P420209	-2.17992	11.68772	-4.4296	.00029	.00206	0.157635	Down
ENPP6	A_24_P397255	-2.17673	4.95395	-9.3829	1.49E-08	3.13E-06	9.91436	Down
ITPKA	A_23_P65918	-2.08495	9.038364	-13.6401	3.03E-11	1.74E-07	15.83023	Down
NEU4	A_23_P218626	-2.05078	6.977062	-3.75447	.001348	.006688	-1.34984	Down
ATP6V0D2	A_23_P146146	-1.95239	7.824694	-4.93318	9.31E-05	.000902	1.278452	Down
PAPSS2	A_24_P940166	-1.92643	10.67104	-6.61756	2.52E-06	7.25E-05	4.860152	Down
ALDH1A1	A_23_P83098	-1.9191	10.55847	-6.36025	4.28E-06	.000103	4.335256	Down
GPT	A_33_P3301871	-1.89279	9.164379	-5.79595	1.41E-05	.000235	3.15381	Down
GATM	A_23_P129064	-1.86222	7.569451	-5.80059	1.39E-05	.000233	3.163685	Down
ALPI	A_23_P337658	-1.85514	9.759881	-6.19711	6.01E-06	.000129	3.997879	Down
LIPC	A_23_P88559	-1.84981	5.399447	-6.90361	1.41E-06	5.07E-05	5.432994	Down
MAOB	A_23_P85015	-1.82724	7.900745	-5.54857	2.40E-05	.000336	2.623745	Down
MAOA	A_33_P3242543	-1.82719	12.43401	-7.59185	3.69E-07	2.04E-05	6.763886	Down
GSTA2	A_23_P214300	-1.75267	6.511859	-4.11049	.000599	.003562	-0.55616	Down
CYP21A2	A_23_P156708	-1.74156	9.308628	-5.91449	1.09E-05	.000196	3.405286	Down
ACADS	A_23_P65022	-1.72155	8.265792	-5.51169	2.60E-05	.000356	2.54414	Down
B3GALT1	A_23_P108564	-1.7101	8.152862	-10.5053	2.44E-09	1.39E-06	11.67164	Down
PLCE1	A_23_P35617	-1.70467	8.920569	-7.94366	1.90E-07	1.39E-05	7.418101	Down
GPAT3	A_33_P3232269	-1.69172	6.957301	-7.44808	4.86E-07	2.45E-05	6.491445	Down
PLA2G10	A_21_P0011456	-1.68911	11.48101	-6.44212	3.61E-06	9.12E-05	4.503244	Down
CYP2C18	A_23_P52480	-1.66286	8.061292	-2.87503	.009717	.030661	-3.24928	Down
NAT2	A_23_P31798	-1.65294	9.657951	-3.08749	.006078	.021419	-2.80346	Down
MTM1	A_33_P3389168	-1.6398	6.823035	-5.70227	1.72E-05	.000268	2.953908	Down
UGP2	A_23_P253046	-1.61599	11.63809	-8.49632	6.94E-08	7.43E-06	8.410414	Down
GPX3	A_33_P3369371	-1.6126	12.76248	-6.3051	4.80E-06	.00011	4.2216	Down
GALNT16	A_23_P76749	-1.61098	8.78124	-4.2653	.000421	.002747	-0.20987	Down
TST	A_23_P29248	-1.5836	13.57648	-7.39015	5.43E-07	2.59E-05	6.380831	Down
CMBL	A_33_P3251751	-1.55357	5.381922	-3.76713	.00131	.006553	-1.32174	Down
HSD17B6	A_23_P25030	-1.5529	7.421588	-4.86421	.000109	.001007	1.125726	Down
EPHX2	A_23_P8834	-1.54817	11.46242	-6.30679	4.78E-06	.00011	4.225075	Down
SELENBP1	A_23_P86021	-1.54512	13.21859	-4.82333	.000119	.001079	1.035074	Down
CYP2A7	A_23_P27528	-1.5394	4.869589	-2.72535	.013458	.039626	-3.55606	Down
ACAA2	A_23_P89799	-1.51965	12.98188	-9.31305	1.68E-08	3.34E-06	9.799691	Down
AMY1A	A_23_P23611	-1.51527	8.436707	-5.65771	1.89E-05	.000286	2.858464	Down
GCNT2	p40927_v4	-1.50393	5.114886	-6.21477	5.79E-06	.000125	4.034566	Down
ENTPD3	A_23_P212469	-1.47203	4.729804	-4.08509	.000634	.003728	-0.61294	Down
CA8	A_23_P83838	-1.46394	6.556976	-4.15611	.00054	.003303	-0.45414	Down
CKMT1A	A_23_P163227	-1.44002	12.62216	-4.98424	8.31E-05	.000829	1.39129	Down
GSTA5	A_23_P93141	-1.4115	5.323562	-3.76883	.001305	.006535	-1.31796	Down
CD38	A_23_P167328	-1.37248	9.027194	-3.25834	.004148	.015938	-2.43763	Down
ACACB	A_33_P3334220	-1.36824	9.127679	-9.67079	9.27E-09	2.36E-06	10.38025	Down
PIK3CG	A_33_P3304170	-1.35996	9.325257	-4.17762	.000514	.003188	-0.40601	Down
PDE9A	RNA95481|RNS_563_153	-1.33751	9.01096	-4.56868	.000211	.00164	0.468291	Down
ADA2	A_33_P3262635	-1.33158	8.878377	-4.49158	.000252	.00186	0.296149	Down
MGAM2	p44257_v4	-1.32733	12.9453	-3.8462	.001094	.005695	-1.14597	Down
AKR1B1	A_23_P258190	-1.31535	10.79434	-6.33437	4.51E-06	.000107	4.281963	Down
GSTM4	A_33_P3410351	-1.30893	11.24552	-5.59171	2.18E-05	.000316	2.71667	Down
GCNT4	A_33_P3370434	-1.28693	7.088467	-4.56486	.000213	.001652	0.459751	Down
UGT2B4	A_23_P386912	-1.28426	4.398431	-2.59476	.017812	.049426	-3.81791	Down
GALNT12	A_23_P415652	-1.27393	11.6267	-6.11394	7.15E-06	.000147	3.824541	Down
OTC	A_23_P257355	-1.2632	7.390631	-2.90387	.009121	.029182	-3.18944	Down
NAT1	A_23_P95594	-1.26145	11.09693	-4.60445	.000195	.001544	0.548081	Down
NUDT12	A_23_P259090	-1.25695	7.696304	-6.91057	1.40E-06	5.02E-05	5.446789	Down
GALNT7	A_33_P3414012	-1.25513	10.06272	-5.26364	4.47E-05	.000527	2.005149	Down
ENPP3	A_23_P404536	-1.22812	8.893336	-2.6872	.014613	.0423	-3.63315	Down
GPX6	A_33_P3418414	-1.22188	6.518854	-4.43797	.000284	.002035	0.176339	Down
ADH4	A_23_P30098	-1.22166	6.415406	-3.03294	.006861	.023439	-2.91896	Down
PTGS1	A_23_P216966	-1.21887	7.753812	-3.26918	.004048	.015635	-2.41424	Down
AKR1C1	A_33_P3403708	-1.21865	6.633322	-4.06499	.000664	.003862	-0.65787	Down
KMO	A_24_P77082	-1.21718	6.867166	-4.69249	.00016	.001336	0.744255	Down
AOC3	A_23_P426305	-1.21119	9.706971	-4.17031	.000522	.003225	-0.42236	Down
MOCS1	A_32_P52785	-1.20917	9.948793	-4.9704	8.57E-05	.000849	1.360719	Down
LPCAT4	A_23_P49009	-1.1993	10.6136	-7.36396	5.72E-07	2.66E-05	6.330675	Down
SMPD1	A_23_P203488	-1.1915	10.14894	-6.0152	8.81E-06	.000169	3.6176	Down
SDR16C5	A_24_P317708	-1.18969	4.720402	-3.09018	.006041	.021327	-2.79773	Down
BHMT2	A_24_P231829	-1.18938	6.695508	-3.6504	.001709	.008063	-1.58036	Down
CA13	A_23_P381714	-1.17945	6.724669	-5.20565	5.08E-05	.000578	1.878292	Down
ATP5PF	A_32_P310335	-1.17603	9.794425	-6.06872	7.87E-06	.000156	3.729928	Down
MGST3	A_23_P51548	-1.1747	10.08572	-8.37511	8.63E-08	8.54E-06	8.196453	Down
ACSS2	A_23_P210900	-1.17194	12.05968	-5.51086	2.60E-05	.000356	2.542345	Down
FMO2	A_33_P3275702	-1.16608	5.803488	-3.22462	.004474	.016895	-2.51029	Down
AHCYL2	A_32_P228501	-1.16198	10.84215	-8.65227	5.26E-08	6.46E-06	8.682715	Down
PRDX6	A_23_P983	-1.15262	12.87092	-9.35426	1.57E-08	3.23E-06	9.86743	Down
PAFAH2	A_24_P71153	-1.14611	9.120505	-6.21414	5.80E-06	.000125	4.033253	Down
HADH	A_23_P167227	-1.13041	11.60912	-5.87225	1.19E-05	.000209	3.315873	Down
MGAT4A	A_23_P28507	-1.10686	11.80917	-7.2593	7.01E-07	3.10E-05	6.129234	Down
COX6B2	A_23_P78571	-1.08905	8.763101	-4.2373	.000448	.002871	-0.27251	Down
SQOR	A_23_P3221	-1.08477	12.51951	-7.04613	1.07E-06	4.13E-05	5.714118	Down
ETNK1	A_33_P3351566	-1.08338	9.599901	-3.23189	.004402	.016696	-2.49464	Down
MOGAT3	A_23_P317796	-1.08264	10.45688	-3.43168	.002806	.011785	-2.0612	Down
PDE1C	A_24_P206328	-1.07959	7.198895	-5.13229	5.98E-05	.000648	1.717371	Down
SMPD3	A_33_P3397180	-1.07875	8.848652	-4.49177	.000252	.00186	0.296559	Down
B3GALT2	A_33_P3286958	-1.07369	8.496149	-5.87169	1.20E-05	.000209	3.314682	Down
PDE6B	A_21_P0014618	-1.07268	6.250138	-5.78154	1.45E-05	.00024	3.123119	Down
HADHB	A_23_P79703	-1.07142	12.00884	-9.87868	6.60E-09	1.96E-06	10.71005	Down
MBOAT1	A_23_P408996	-1.0689	10.02491	-5.45147	2.96E-05	.000394	2.413867	Down
MAN1A1	RNA147157|p0261_imsncRNA438	-1.06147	7.216949	-7.7558	2.70E-07	1.68E-05	7.070952	Down
BDH2	A_24_P80500	-1.05783	6.643607	-2.89401	.009321	.029676	-3.20992	Down
ACOT1	A_24_P161036	-1.0498	9.505771	-4.68309	.000163	.001357	0.72332	Down
UGT2B10	A_23_P7342	-1.04659	10.49535	-3.62067	.001828	.00848	-1.64604	Down
XYLT1	A_24_P787897	-1.03478	8.158611	-3.76904	.001304	.006533	-1.31751	Down
PDE3B	A_33_P3240018	-1.02361	9.566102	-3.37003	.003226	.013119	-2.19562	Down
CYP2U1	A_33_P3252605	-1.02139	7.48918	-4.806	.000124	.001106	0.996607	Down
CDO1	A_23_P30294	-1.01503	4.464436	-3.23976	.004325	.016482	-2.4777	Down
ATP6V1D	A_33_P3387463	-1.00974	9.539336	-10.0547	4.97E-09	1.69E-06	10.98508	Down
MBOAT2	A_24_P114255	-0.99808	7.716797	-3.78431	.00126	.006362	-1.28359	Down
CDA	A_23_P34597	-0.98903	11.97926	-2.95798	.008097	.026567	-3.07655	Down
ENPP2	A_23_P94338	-0.9858	9.381715	-2.65419	.015687	.044735	-3.69947	Down
CYP27A1	A_33_P3361422	-0.98431	8.453964	-2.67174	.015106	.043424	-3.66424	Down
PDE8A	A_33_P3244951	-0.98421	9.325412	-6.31132	4.73E-06	.000109	4.234442	Down
ST6GALNAC3	A_23_P416711	-0.97462	6.410868	-4.66322	.000171	.001399	0.679077	Down
IMPA1	A_24_P183864	-0.97252	10.51218	-5.00915	7.86E-05	.000792	1.446269	Down
CDS1	A_23_P7250	-0.97244	9.947145	-6.18907	6.11E-06	.00013	3.981148	Down
DAO	A_23_P139635	-0.9711	5.131749	-3.31089	.003686	.014547	-2.32402	Down
PIP5K1B	A_32_P465742	-0.97044	10.69459	-5.385	3.42E-05	.000435	2.269613	Down
ARG2	p42844_v4	-0.96299	5.137903	-4.62599	.000186	.001486	0.596104	Down
MGLL	A_32_P88240	-0.9566	7.196389	-7.37837	5.56E-07	2.62E-05	6.358282	Down
PLCG2	A_23_P106675	-0.94916	7.839206	-3.54953	.002149	.009559	-1.8028	Down
CAT	A_23_P105138	-0.9478	11.69661	-5.62119	2.05E-05	.000303	2.780059	Down
ALPP	A_23_P79587	-0.93556	5.538592	-4.7634	.000136	.001185	0.901971	Down
ALDH3A2	A_33_P3336622	-0.93413	9.362768	-5.20053	5.14E-05	.000582	1.867078	Down
HGD	A_23_P250164	-0.93075	8.414698	-3.59926	.001919	.008789	-1.69326	Down
B4GALNT3	A_23_P403443	-0.92571	8.818165	-4.78223	.000131	.001148	0.943812	Down
ATP5F1A	A_24_P245358	-0.91882	12.8982	-5.05815	7.05E-05	.000732	1.5543	Down
SUCLG2	A_33_P3636590	-0.91853	12.30562	-6.43828	3.64E-06	9.12E-05	4.495384	Down
HADHA	A_24_P242688	-0.91687	11.96042	-7.79174	2.53E-07	1.60E-05	7.137763	Down
DGKA	A_23_P105307	-0.91578	12.25564	-3.95173	.00086	.004729	-0.9108	Down
FMO4	A_23_P305507	-0.91074	9.825161	-4.41611	.000299	.002106	0.127475	Down
PLA2G4F	A_24_P147169	-0.91011	8.666782	-4.93176	9.34E-05	.000904	1.275318	Down
LARGE1	RNA147237|p0341_imsncRNA762	-0.90385	9.462658	-4.03335	.000714	.004077	-0.72858	Down
PGM1	A_23_P52031	-0.90308	11.82112	-5.34547	3.73E-05	.000463	2.183636	Down
AUH	A_23_P20852	-0.90126	9.27831	-2.72786	.013385	.039452	-3.55097	Down
ATP6V0D1	A_23_P54636	-0.89405	8.173407	-6.02128	8.70E-06	.000168	3.630386	Down
HACD4	A_23_P83175	-0.89217	9.034161	-3.22756	.004445	.016819	-2.50396	Down
PANK3	A_33_P3211423	-0.89165	11.3893	-6.1301	6.91E-06	.000143	3.858281	Down
AZIN2	A_24_P11462	-0.88812	8.459265	-2.75477	.012628	.037706	-3.49629	Down
CHPT1	A_23_P105571	-0.88616	10.5294	-4.26752	.000419	.002736	-0.2049	Down
ACADVL	A_23_P207650	-0.88445	13.32788	-5.42102	3.17E-05	.000415	2.347845	Down
AMT	A_23_P257164	-0.8797	8.809956	-4.37821	.000325	.002256	0.042736	Down
MLYCD	A_19_P00317553	-0.86849	9.462098	-6.77789	1.82E-06	5.90E-05	5.182635	Down
ACADM	A_23_P96761	-0.86832	11.62882	-3.47258	.002558	.010956	-1.97172	Down
ADCY5	p40173_v4	-0.86725	7.062551	-4.88458	.000104	.000974	1.170873	Down
RIMKLA	A_23_P349406	-0.86008	9.922611	-2.67001	.015163	.043554	-3.66772	Down
MPI	A_23_P60579	-0.85865	10.03992	-4.39672	.000312	.002176	0.084108	Down
ACAT1	A_33_P3253975	-0.85333	10.25275	-4.9153	9.69E-05	.00093	1.238882	Down
ACOX3	A_23_P316381	-0.85176	10.45667	-5.29476	4.18E-05	.000501	2.073097	Down
PLCD3	A_33_P3310226	-0.83764	10.66806	-4.5033	.000245	.001833	0.322329	Down
NNT	A_24_P174775	-0.82803	7.937086	-3.62721	.001801	.008385	-1.63158	Down
B3GNT5	A_23_P18372	-0.82684	10.04085	-3.65009	.00171	.008064	-1.58105	Down
ACOT4	A_23_P14515	-0.82398	7.38384	-3.36632	.003253	.013198	-2.2037	Down
PLD1	A_33_P3334843	-0.81488	8.3032	-4.49692	.000249	.001843	0.308064	Down
ARSA	A_33_P3229725	-0.80668	10.95945	-5.64675	1.94E-05	.000292	2.834943	Down
TPK1	A_33_P3423530	-0.79882	9.266724	-4.56394	.000214	.001653	0.45771	Down
CYP3A7	A_33_P3318117	-0.79534	8.718912	-3.04364	.0067	.023022	-2.89636	Down
ABO	A_33_P3266550	-0.7898	12.22068	-4.18029	.000511	.003173	-0.40004	Down
ALDH6A1	A_23_P76983	-0.78778	9.120224	-6.55494	2.86E-06	7.88E-05	4.733251	Down
UAP1	A_23_P160460	-0.78594	10.81044	-6.44057	3.62E-06	9.12E-05	4.500075	Down
COQ6	A_23_P65584	-0.78347	9.366381	-6.13013	6.91E-06	.000143	3.858344	Down
SEPHS2	A_23_P146798	-0.78317	12.60216	-4.01501	.000744	.004213	-0.76954	Down
MTMR8	A_23_P84995	-0.77762	8.259032	-2.93332	.008549	.027717	-3.12809	Down
CYP3A5	A_33_P3249746	-0.77366	10.76191	-4.45417	.000274	.001981	0.212547	Down
GBGT1	A_23_P146576	-0.77262	8.629942	-3.70449	.001511	.007302	-1.46066	Down
RFK	A_23_P216708	-0.76437	9.958526	-3.69905	.00153	.007375	-1.47272	Down
PLCB2	A_33_P3260614	-0.76425	9.346559	-2.99996	.00738	.024781	-2.98847	Down
DOLPP1	A_23_P386764	-0.76407	9.549525	-5.17572	5.43E-05	.000607	1.812684	Down
ALAD	A_23_P217114	-0.76265	10.83027	-5.8139	1.35E-05	.000229	3.192001	Down
MPST	A_23_P132285	-0.75827	11.68621	-4.55942	.000216	.001665	0.447627	Down
IDNK	A_23_P123732	-0.75753	8.789317	-4.57294	.000209	.001626	0.477799	Down
CERS4	A_23_P153867	-0.75362	6.359532	-4.09946	.000614	.003636	-0.58083	Down
UQCR10	A_23_P68866	-0.75136	10.8633	-5.02459	7.59E-05	.000775	1.48033	Down
ASL	A_23_P26223	-0.75051	11.24641	-4.34084	.000354	.002406	-0.04085	Down
ASAH1	A_33_P3284463	-0.74933	12.01576	-4.74172	.000143	.001231	0.853774	Down
PDE7B	A_33_P3266025	-0.7469	3.994376	-3.85115	.001082	.00565	-1.13494	Down
PDE2A	A_23_P401106	-0.745	5.776012	-4.21648	.00047	.002976	-0.31909	Down
GALM	A_24_P212539	-0.73536	11.46717	-7.16772	8.38E-07	3.49E-05	5.951683	Down
ST8SIA1	p42426_v4	-0.72307	7.433576	-3.02941	.006915	.023574	-2.92642	Down
ACER3	A_23_P203665	-0.71497	11.02197	-4.53956	.000226	.001724	0.403297	Down
PIP4K2A	A_33_P3394065	-0.71311	6.978154	-4.05419	.000681	.003932	-0.68201	Down
ACSM6	A_33_P3400253	-0.70941	5.028972	-3.80073	.001213	.006186	-1.24711	Down
MAN1C1	A_23_P103601	-0.70769	8.000836	-2.84922	.010281	.032106	-3.30264	Down
ATP5F1B	RNA33687|snoRNA_scaRNA_283_75	-0.70747	10.55086	-6.14926	6.64E-06	.000139	3.898263	Down
NDUFB9	A_33_P3354256	-0.70396	9.518557	-4.05975	.000672	.003897	-0.66959	Down
ACADSB	A_23_P158570	-0.70193	8.632056	-2.76246	.012419	.037218	-3.48062	Down
ST3GAL5	p37201_v4	-0.69766	5.263404	-3.54236	.002184	.009679	-1.81858	Down
MAN2A1	A_23_P360769	-0.69408	11.29166	-3.79817	.00122	.006217	-1.25279	Down
GBA2	A_24_P341187	-0.69191	11.10618	-5.9165	1.09E-05	.000196	3.409542	Down
DGLUCY	A_23_P48771	-0.69188	10.54653	-3.47792	.002528	.010861	-1.96003	Down
BTD	A_24_P217365	-0.68447	8.220816	-4.68942	.000161	.001342	0.737422	Down
FAHD1	A_32_P75902	-0.67864	9.806943	-4.86798	.000108	.000999	1.134081	Down
SYNJ1	A_23_P324718	-0.67833	7.274129	-3.1753	.004997	.018407	-2.61616	Down
SUCLG1	A_23_P79545	-0.6745	12.03001	-3.98406	.000799	.004451	-0.83865	Down
BPNT1	A_23_P104046	-0.673	8.86608	-4.24787	.000438	.002824	-0.24884	Down
CDS2	A_24_P271363	-0.67272	9.471116	-4.7856	.00013	.001142	0.951306	Down
ABAT	A_33_P3268487	-0.67013	8.12548	-3.477	.002533	.010876	-1.96203	Down
HAGH	A_33_P3333488	-0.66824	9.552575	-5.10078	6.41E-05	.000682	1.648113	Down
PDHA1	A_23_P251095	-0.66564	9.431063	-3.39814	.003028	.012523	-2.1344	Down
MPPE1	A_23_P27285	-0.66225	9.201225	-4.4909	.000252	.001862	0.294623	Down
GALNT11	RNA96152|RNS_1234_64	-0.65675	6.775279	-5.59105	2.19E-05	.000316	2.715252	Down
NADSYN1	A_23_P35848	-0.655	12.10039	-3.79549	.001228	.00624	-1.25875	Down
NDUFS1	A_23_P131365	-0.64629	12.1623	-5.55934	2.34E-05	.00033	2.646961	Down
ACAD8	A_23_P47426	-0.64525	8.007872	-4.87183	.000107	.000995	1.142628	Down
NANS	A_33_P3291776	-0.64024	12.29228	-3.71337	.001481	.007194	-1.441	Down
IMPA2	A_23_P50081	-0.63991	10.75461	-3.3514	.003364	.01355	-2.23614	Down
HYI	A_23_P200976	-0.63648	9.804949	-4.68953	.000161	.001342	0.737668	Down
KMT5B	A_33_P3424067	-0.63569	9.484611	-4.04294	.000698	.004006	-0.70715	Down
UQCRC2	A_23_P118002	-0.63505	12.93155	-4.23135	.000455	.0029	-0.28581	Down
PIGN	A_24_P193943	-0.634	8.336371	-3.03817	.006781	.023237	-2.90791	Down
MMUT	A_23_P400235	-0.63154	10.34847	-2.88972	.009409	.029886	-3.21883	Down
KMT2E	A_33_P3277407	-0.62949	11.69886	-4.79979	.000126	.001117	0.982809	Down
DERA	A_23_P25253	-0.62778	10.17765	-5.01759	7.71E-05	.000783	1.464891	Down
HMGCLL1	A_32_P49508	-0.62632	5.681939	-2.66934	.015185	.043577	-3.66907	Down
ENTPD4	A_33_P3397486	-0.62548	10.41745	-5.1545	5.69E-05	.000626	1.766141	Down
PCBD2	RNA143468|tRNA_385_71	-0.62085	12.02254	-3.56848	.002058	.009278	-1.76109	Down
ARG1	A_33_P3352382	-0.61731	5.964446	-2.745	.012898	.038339	-3.51616	Down
ALOX12	A_33_P3328445	-0.61575	5.142321	-3.12961	.005533	.019941	-2.71381	Down
SDHD	A_23_P138967	-0.61438	11.30378	-4.12336	.000581	.003488	-0.52739	Down
GSTM2	A_23_P115407	-0.61404	13.13531	-3.00013	.007378	.024775	-2.9881	Down
AOX1	A_23_P154037	-0.61365	7.507271	-2.84018	.010487	.032599	-3.32129	Down
NDST4	A_23_P136371	-0.60907	4.219856	-2.93029	.008607	.027869	-3.13441	Down
FUT2	A_24_P942969	-0.6044	10.59931	-3.42666	.002839	.011897	-2.07216	Down
KMT2C	A_33_P3366859	-0.60313	11.1487	-3.78183	.001267	.006389	-1.28909	Down
CYP2R1	A_23_P202860	-0.60066	9.703134	-3.92022	.000924	.004995	-0.98108	Down
PNLIPRP3	A_33_P3368193	-0.59951	4.063199	-2.63514	.01634	.046149	-3.73758	Down
GSTZ1	A_23_P106204	-0.59594	8.061256	-3.11366	.005734	.020496	-2.74779	Down
PNPO	A_33_P3243175	-0.59215	12.43405	-4.40783	.000304	.002133	0.10895	Down
DLST	A_23_P205697	-0.58624	10.51282	-3.9217	.000921	.004986	-0.97779	Down
AOC1	RNA95517|RNS_599_149	-0.58619	10.39325	-2.83666	.010567	.032767	-3.32854	Down
PAFAH1B1	A_23_P118888	-0.58568	11.70086	-5.27618	4.35E-05	.000517	2.032531	Down
NMRK1	p41788_v4	-0.57966	6.777982	-4.09752	.000617	.003647	-0.58517	Down
GSR	A_23_P146084	-0.57555	12.30612	-3.06279	.006421	.022299	-2.85583	Down
SDHA	A_23_P250035	-0.57233	12.26396	-3.88908	.000992	.00528	-1.05049	Down
IPMK	A_23_P380371	-0.56901	9.921534	-3.77779	.001278	.006435	-1.29806	Down
SGPP2	A_24_P941217	-0.56854	9.70651	-3.75525	.001346	.006685	-1.34812	Down
NMNAT1	A_33_P3215193	-0.56769	9.756203	-2.96137	.008037	.026409	-3.06946	Down
SIRT6	A_23_P208847	-0.5635	9.072564	-3.47909	.002521	.010836	-1.95746	Down
SCP2	RNA147153|p0257_imsncRNA419	-0.55952	10.3243	-5.65055	1.92E-05	.00029	2.843108	Down
ALDH7A1	A_33_P3330503	-0.55617	8.181922	-2.63104	.016484	.046487	-3.74575	Down
PIK3CD	A_33_P3353941	-0.54788	7.11061	-2.64104	.016135	.045677	-3.72579	Down
PDE4D	A_33_P3389653	-0.54334	7.921093	-4.26309	.000423	.002758	-0.21479	Down
GGT6	A_23_P66767	-0.54177	10.38265	-3.63651	.001763	.008264	-1.61106	Down
PDE1A	A_24_P208436	-0.54	7.725237	-2.64361	.016046	.045503	-3.72064	Down
GGT7	A_23_P57236	-0.53147	8.890434	-2.9785	.007739	.025707	-3.03355	Down
NDUFV1	A_33_P3317406	-0.53057	12.67437	-3.04428	.00669	.023002	-2.89501	Down
PIGB	A_23_P77174	-0.52881	8.200877	-2.86512	.00993	.03124	-3.26978	Down
NUDT9	A_23_P94860	-0.52696	10.10819	-3.73835	.001399	.006888	-1.38562	Down
B3GALT4	A_23_P422071	-0.52604	9.07751	-4.1268	.000577	.003466	-0.51969	Down
AK5	A_33_P3307247	-0.52365	4.399321	-3.5863	.001977	.008998	-1.72183	Down
ALDH2	A_23_P36753	-0.5041	12.71495	-2.88425	.009522	.030159	-3.23016	Down
ALAS1	A_24_P191588	-0.50285	10.94569	-3.20362	.00469	.01752	-2.55543	Down
NUDT16	A_23_P310560	-0.50243	8.09093	-3.9011	.000965	.00517	-1.02369	Down
COQ7	A_24_P398972	-0.50093	8.206341	-4.26672	.000419	002739	-0.20667	Down
MTHFR	A_23_P404045	-0.50084	10.89013	-3.37633	.00318	.012981	-2.18191	Down
